# Genomic Analyses Revealed the Genetic Difference and Potential Selection Genes of Growth Traits in Two Duroc Lines

**DOI:** 10.3389/fvets.2021.725367

**Published:** 2021-09-07

**Authors:** Desen Li, Min Huang, Zhanwei Zhuang, Rongrong Ding, Ting Gu, Linjun Hong, Enqin Zheng, Zicong Li, Gengyuan Cai, Zhenfang Wu, Jie Yang

**Affiliations:** ^1^College of Animal Science and National Engineering Research Center for Breeding Swine Industry, South China Agricultural University, Guangzhou, China; ^2^Lingnan Guangdong Laboratory of Modern Agriculture, Guangzhou, China

**Keywords:** Duroc, genetic difference, growth trait, *F*_ST_, XP-EHH

## Abstract

Duroc pigs are famous for their high growth rate, feed conversion efficiency, and lean meat percentage. Given that they have been subjected to artificial selection and breeding in multiple countries, various lines with obvious differences in production performance have formed. In this study, we genotyped 3,770 American Duroc (AD) pigs and 2,098 Canadian Duroc (CD) pigs using the GeneSeek Porcine SNP50 Beadchip to dissect the genetic differences and potential selection genes of growth traits in these two Duroc pig lines. Population structure detection showed that there were significant genetic differences between the two Duroc pig lines. Hence, we performed *F*_ST_ and cross-population extended haplotype homozygosity (XP-EHH) analyses between the two lines. As a result, we identified 38 annotated genes that were significantly enriched in the gland development pathway in the AD line, and 61 annotated genes that were significantly enriched in the immune-related pathway in the CD line. For three growth traits including backfat thickness (BFT), loin muscle depth (LMD), and loin muscle area (LMA), we then performed selection signature detection at 5 and 10% levels within the line and identified different selected regions and a series of candidate genes that are involved in lipid metabolism and skeletal muscle development or repair, such as *IRX3, EBF2, WNT10B, TLR2, PITX3*, and *SGCD*. The differences in selected regions and genes between the two lines may be the cause of the differences in growth traits. Our study suggests significant genetic differences between the AD and CD lines, which provide a theoretical basis for selecting different Duroc lines as sires for different needs.

## Introduction

Lean pigs have been selected and bred in various countries for a long time and have formed distinctive lines, such as English Large White and French Large White. Since the 1980s, China introduced Duroc pigs from America (American line), Canada (Canadian line), Denmark (Danish line), China Taiwan (Taiwan line), and Japan (Japanese line). Among these Duroc lines, the Taiwan line has the characteristics of beautiful body shape, rough feeding resistance, and strong disease resistance, but the growth rate is slow during the late fattening period. The American line has a higher growth rate, stress resistance, and lean meat percentage than the Taiwan line. The Canadian line is well-known for its high average daily gain, rich intramuscular fat (IMF) content, and excellent meat quality (1).

Quan et al. assessed the carcass traits of American Duroc × (Landrace × Yorkshire) three-way cross hybrid (ADLY) pigs and Taiwan Duroc × (Landrace × Yorkshire) three-way cross hybrid (TDLY) pigs and found that the lean meat percentage of ADLY pigs was better than that of TDLY pigs (ADLY: 57.39 vs. TDLY: 55.27%) (*p* < 0.01), while the live mass (ADLY: 104.06 vs. TDLY: 110.02 kg), carcass mass (ADLY: 88.31 vs. TDLY: 94.14 kg), loin muscle depth (LMD) (ADLY: 51.16 vs. TDLY: 54.61 cm), and carcass italic length (ADLY: 84.16 vs. TDLY: 86.01 cm) of TDLY were better than those of ADLY (*p* < 0.01) (2). Zhuang et al. conducted genome-wide association studies (GWAS) for loin muscle area (LMA) and LMD in American and Canadian Duroc pigs and identified 75 significantly associated SNPs, of which a 283-kb region on chromosome 7 was a pleiotropic quantitative trait loci (QTL) that affected both traits. Among these 75 SNPs, the ALGA0040260 marker was the key SNP for the QTL and explained 1.77 and 2.48% of the phenotypic variance in LMA and LMD, respectively (3). In addition, Zhuang et al. also performed GWAS for teat number in American and Canadian Duroc pigs and detected a QTL on chromosome 7 with marker rs692640845 explaining 8.68% of the phenotypic variance in the Canadian Duroc (4). Although these studies conducted GWAS for different lines of Duroc pigs and identified different candidate genes and QTLs, they did not uncover the genetic differences between different Duroc lines.

For different production needs, different lines of pigs have formed their own characteristics through artificial selection. Animals are usually selected for certain traits, and the internal mechanism is the selection of genes. The selection signature detection can reveal potential selection genes, which is of great significance for understanding the evolution of species and identifying genes for economic traits. Ma et al. performed the cross-population extended haplotype homozygosity (XP-EHH) and *F*_ST_ to detect trait-specific selection signatures by making backfat thickness (BFT) gradient differential population pairs in Yorkshire pigs, and identified that a number of genes were associated with fat metabolism, such as *OSBPL8, ASAH2, GBE1*, and *ABL1* (5). Kim et al. used the Duroc pigs that were sampled from the sixth generation of a selection experiment for IMF to divide the high and low IMF groups and to conduct selection signature detection, and a total of 16 consensus regions were obtained using the three methods [including *F*_ST_, the integrated haplotype score (IHS), and the standardized score of the ratio of extended haplotype homozygosity (Rsb)] (6). The above studies show the feasibility of dividing the phenotypic gradient groups within the population for selective signature detection, but there is no relevant research on the use of this method in different lines of Duroc.

Therefore, in this study, we used Porcine SNP50 Beadchip to genotype 3,770 America Duroc (AD) and 2,098 Canadian Duroc (CD) pigs and carried out selection signature detection between the two lines. Besides, for the same line, we also performed selection signature detection through dividing extreme phenotypic groups according to the estimated breeding values (EBV) ranking of BFT, LMD, and LMA, and then have identified the selected regions and genes in different lines to reveal the potential genetic mechanisms that caused the differences in growth traits.

## Materials and Methods

### Ethics Statement

All animals used in this study met the guidelines for the care and use of experimental animals established by the Ministry of Agriculture of China. The whole of this study was approved by the ethics committee of South China Agriculture University (SCAU, Guangzhou, China), and written informed consent was obtained prior to data collection from Wens Foodstuff Group Co., Ltd. (Guangdong, China). There was no use of human participants, data, or tissues.

### Sample collection, SNP Genotyping, and Phenotype Detection

A total of 3,770 American Duroc pigs and 2,098 Canadian Duroc pigs were genotyped using the GeneSeek Porcine SNP50 Beadchip in this study. All pigs in the two populations sustained uniform feeding conditions, fine fodder, and consistent management during the fattening period from 30 to 100 kg live weight to minimize the impact of non-genetic factors. The details of sample collection, DNA extraction, SNP genotyping, and recording of LMA and LMD were described by Zhuang et al. (3). In addition, BFT was obtained by measuring the thickness of backfat between the 10th and 11th rib of the pigs at the weight of 100 ± 5 kg using an Aloka 500 V SSD B ultrasound (Corometrics Medical Systems, USA). The three phenotypes were corrected by 100 kg body weight. Quality filtering of genotypes was performed using PLINK v1.9 (7) with the criteria of minor allele frequencies (MAF) >0.01, individual call rate >95%, and SNP call rate >95%. After removing non-autosomal and unmapped SNPs, a total of 39,567 SNPs remained and used in subsequent analyses.

### Population Structure and Estimation of Inbreeding Coefficient

Genetic distance among individuals was calculated via an identity-by-state (IBS) similarity matrix by PLINK v1.9. A neighbor-joining relationship tree (NJ-tree) based on the genetic distance was constructed using PHYLP v3.69 (8) and was visualized using Figtree v1.4 (9). We randomly selected 100 individuals from each of the two lines 10 times and used PLINK v1.9 to estimate linkage disequilibrium decay (LD decay) distance. When *r*^2^ = 0.3, the physical distance was used to identify the range of the annotated genes (10). Runs of homozygosity (ROH) of the two lines were performed using the consecutiveRUNS.run function of the R detectRUNS package (11). The inbreeding coefficient based on ROH (*F*_*ROH*_) was calculated for each individual using the following formula (12):

$$\begin{array}{l} F_{ROH}=\;\frac{\sum_{i}L_{ROH}}{L_{AUTO}} \end{array}$$

where *L*_*ROH*_ is the length of ROH of individual *i*, and *L*_*AUTO*_ is the autosomal genome length covered by the SNPs in this study.

### Estimated Breeding Value Calculation

Additive effect (breeding value), dominance effect, and epistatic effect can affect quantitative traits, among which additive effect can be stably inherited by offspring, which means the EBV can be calculated from phenotypes and parentage. In this study, the EBV of BFT, LMD, and LMA were calculated based on the restricted maximum likelihood (REML) method via the dumai model of DMU software (13). The calculation model is as follows:

$$\begin{array}{l} y\;=\;u+Xb+Za+e \end{array}$$

where *y* is the vector of phenotypic values, *b* is the fixed effects vector, including sex, farm, year, season, and parity (Supplementary Table 1), *a* is the vector of individual random additive effect, *e* is the vector of random residuals, and *X* and *Z* are the structural matrices of *a* and *b*, respectively. For BFT, a single-trait animal model was used to obtain the EBV. A multitrait animal model was used in LMD and LMA because of their strong correlation with muscle. As a result, 50% of EBV of the two traits was taken to obtain the total breeding value (TBV) for the two traits.

### Selection Signature Detection Between the American Duroc Line and the Canadian Duroc Line

To detect the degree of genetic differentiation between the AD and CD lines, *F*_ST_ between the two lines was performed using VCFtools (14). The XP-EHH was operated using the “–xpehh” function of Selscan (15) based on the haplotypes constructed by Beagle (16). The top 1% values were taken as the significant thresholds of *F*_ST_ and XP-EHH, respectively, and the overlapping SNPs of two methods exceeding the thresholds were considered as the selected markers.

### Selection Signature Detection for Genetic Differential in Different Gradients of Growth Traits Within the American Duroc Line and the Canadian Duroc Line

According to the EBV ranking of BFT, LMD, and LMA in the AD and CD lines, the individuals with the top 5% EBV were selected for a group and the bottom 5% of which were selected for the other group, defined as the 5% level. A similar strategy was defined at the 10% level. Then the XP-EHH and the *F*_ST_ were used to detect the selection signature in the two levels for each trait within the two lines. A value of 0.05 was used as the significance threshold for *F*_ST_, and the top 5% was used as the significance threshold for XP-EHH.

### Candidate Gene and Functional Annotation

When *r*^2^ = 0.3, the LD decay distances of the two lines were used as the upstream and downstream ranges of the selected markers to determine the selected regions. We then searched the annotated genes from the selected regions and conducted Gene Ontology (GO) and Kyoto Encyclopedia of Genes and Genomes (KEGG) pathway analysis by Metascape (https://metascape.org/) to identify the candidate genes whose functions are associated with growth traits. In addition, the annotated genes were compared with the pig QTL database (https://www.animalgenome.org/) to identify the genes within the QTL regions for growth traits.

## Results

### Population Structure

Our previous study performed principal component analysis (PCA) between these two Duroc pig populations (3), and the results showed that PC1 divided the AD and CD lines into two obvious groups, and PC2 showed that there was stratification phenomenon in the AD line as well. Then, in this study, the NJ tree showed that the two lines formed two apparently independent branches (Supplementary Figure 1A), and there were multiple lineages within the AD line. PCA and NJ-tree analysis revealed that there was significant genetic differentiation between the AD and CD lines, which implied that there were obvious genetic differences between the two Duroc lines. To avoid the impact of population stratification in the AD line, PCA was conducted for each lineage of the AD line and the CD line according to the NJ tree of the AD line (Supplementary Figure 2). Finally, a total of 1,969 AD line pigs with relatively concentrated clustering and more differentiated with the CD line were selected for subsequent analysis. The results of LD decay analysis showed that the decay rate of the CD line was slower than that of the AD line. When *r*^2^ = 0.3, the average LD decay distances in the AD and CD lines were approximately 150 and 202 kb, respectively (Supplementary Figure 1B). Although the same Porcine SNP50 Beadchip data were utilized for LD decay analysis in the study by Zhuang et al. (4), our study had different population size, analysis methods, and threshold line criteria, and therefore, the LD decay analysis was reperformed in this study rather than directly cited. Besides, the average *F*_*ROH*_ of the AD line was lower than that of the CD line (Supplementary Figure 1C). The results of LD decay and *F*_*ROH*_ indicated a higher degree of inbreeding in the CD line.

### EBV Calculation

We corrected and summarized the statistics for BFT, LMD, and LMA in the AD and CD lines, respectively, and all of these traits were normally distributed as shown in Supplementary Figure 3A. We found that there were significant differences between the two lines in these phenotypes, of which LMD and LMA in the AD line were greater than those in the CD line, while BFT was lower than the latter (Supplementary Figure 3B). Next, the EBV of BFT, LMD, and LMA were calculated separately in the two lines (Supplementary Tables 2, 3).

### Selection Signature Detection Between the American Duroc Line and the Canadian Duroc Line

**Figure 1:**
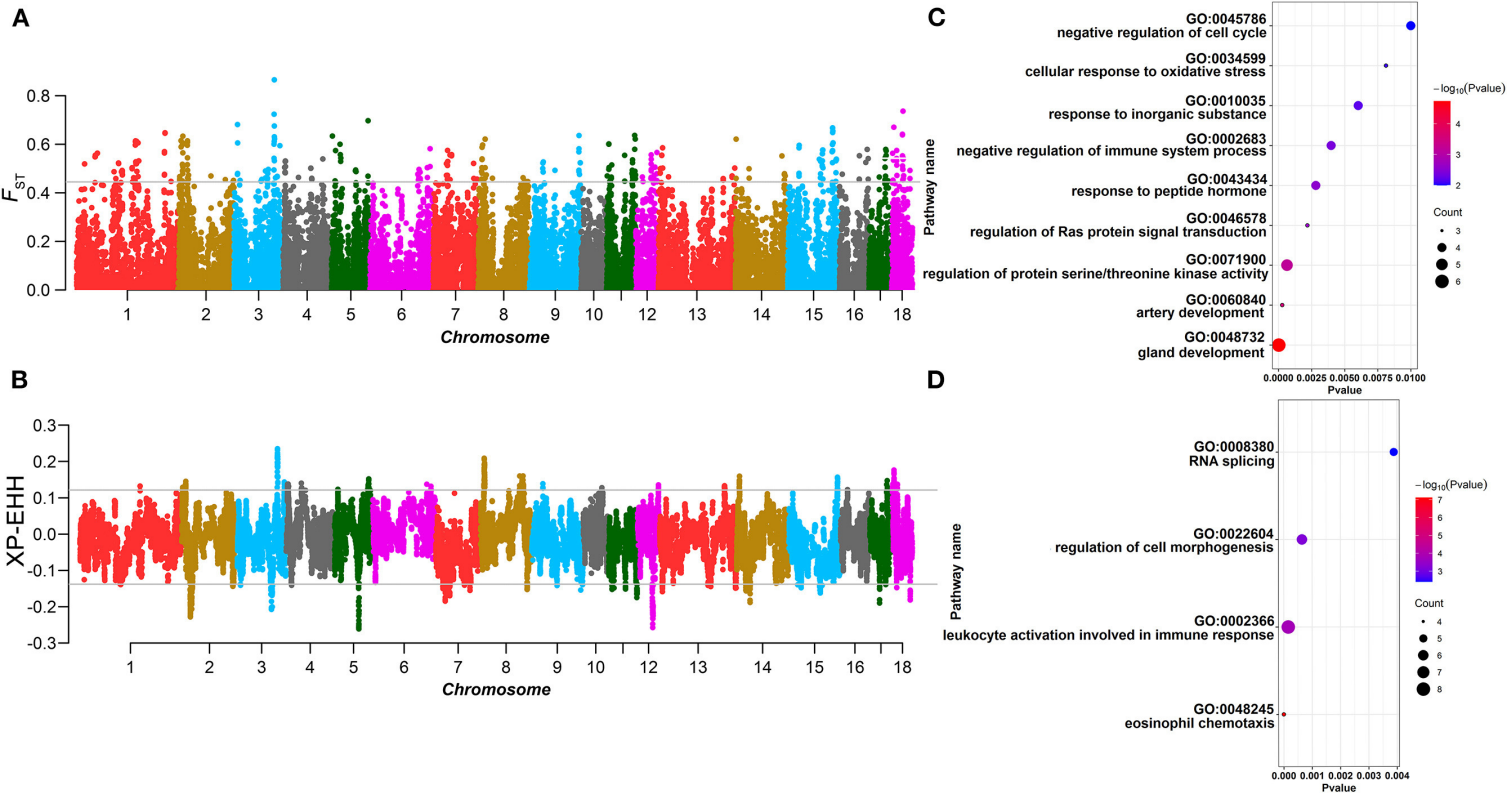
Selection signature detection between the American Duroc (AD) line and the Canadian Duroc (CD) line. **(A)** Manhattan plot of *F*_ST_. The gray line denotes threshold line (*F*_STtop1%_ = 0.44). **(B)** Manhattan plot of XP-EHH. The gray lines denote threshold lines (XP-EHH_AD_ = 0.12, XP-EHH_CD_ = −0.14). **(C,D)** are the bubble chart of Gene Ontology (GO) terms and Kyoto Encyclopedia of Genes and Genomes (KEGG) pathways of the selected genes identified from the AD and CD lines, respectively.

Population structure analysis revealed a large degree of genetic differentiation between the AD and CD lines. Hence, we performed selection signature detection between 1,969 and 2,098 individuals from the AD and CD lines, respectively. In the XP-EHH analysis, the AD line was used as the test population and the CD line as the referencepopulation, which means that the positive values represent recent selection in the AD line, and conversely, the negative values represent that of the CD line (Figure 1B). The top 1% was used as the significant threshold for *F*_ST_ (Figure 1A) and XP-EHH (*F*_STtop1%_ = 0.44, XP-EHH_AD_ = 0.12, and XP-EHH_CD_ = −0.14), and the overlapping significant SNPs in the two statistics were defined as the selected markers. A total of 28 selected markers and 38 annotated genes were identified in the AD line from the 300-kb selected region (150 kb upstream and downstream of the selected SNPs), while a total of 30 selected markers and 61 annotated genes were found in the CD line from the 404-kb selected region (202 kb upstream and downstream of the selected SNPs). GO enrichment and KEGG pathway analysis of the annotated genes showed that the genes were significantly enriched in the gland development pathway (GO: 0048732) in the AD line (Figure 1C). For the CD line, the genes were significantly enriched in immune-related pathways (GO: 0048245, GO: 0002366) (Figure 1D; Supplementary Table 4).

### Selection Signature of Backfat Thickness Within the American Duroc line and the Canadian Duroc Line

#### The 5% Level

**Figure 2:**
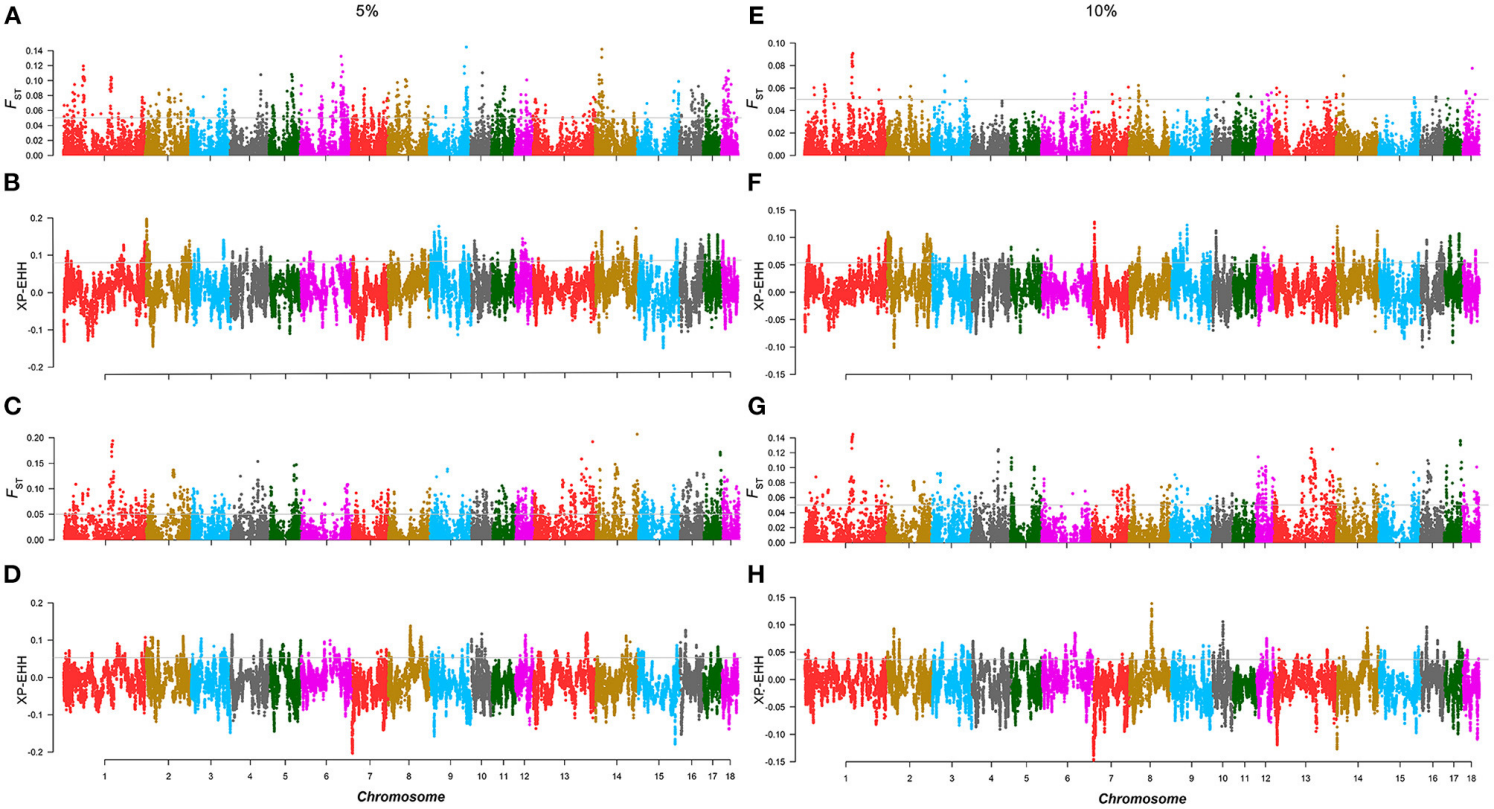
Selection signature detection for backfat thickness (BFT) in the 5 and 10% levels. **(A,C,E,G)** are the Manhattan plots of *F*_*S*T_ of the AD and CD lines in the 5 and 10% level. The gray lines denote threshold lines (*F*_ST_ = 0.05). **(B)**, **(D,F,H)** are the Manhattan plots of XP-EHH of AD and CD lines in the 5 and 10% levels. The gray lines denote threshold lines (XP-EHH_5%AD_ = 0.080, XP-EHH_5%CD_ = 0.053, XP-EHH_10%AD_ = 0.054, XP-EHH_10%CD_ = 0.036).

For the AD line, according to the EBV ranking of BFT, 98 individuals with the top and the bottom EBV values were selected from 1,969 individuals as two groups to carry out *F*_ST_ and XP-EHH. In XP-EHH, the top 5% group was used as the test population, and the bottom 5% group was used as the reference population. The overlapping SNPs that exceeded the significant threshold in both methods (*F*_ST_ > 0.05, XP-EHH_AD_ = 0.08) were regarded as the selected markers in the AD line. A total of 46 selected markers and 35 annotated genes were identified. GO and KEGG enrichment analysis of the annotated genes (Supplementary Figure 4) revealed that genes such as *IRX3* and *EBF2* are involved in lipid metabolism. Similarly, 105 individuals with the top and bottom EBV values were selected from the CD line, respectively, as the two groups to detect the selection signature. A total of 131 selected markers exceeded the thresholds in both *F*_ST_ and XP-EHH (XP-EHH_CD_ = 0.05), and 167 annotated genes were identified. We found that *SAMD4A, DLGAP5, CTSF*, etc. (Figures 2A–D; Table 1) are involved in lipid deposition-related pathways according to GO and KEGG enrichment analysis (Supplementary Figure 5).

**Table 1:** Candidate genes for BFT in the 5% level analysis.

**Level**	**Line**	**Chr**	**Pos (bp)**	**Candidate gene**	**QTL**
5%	AD	6	31,046,098–31,048,822	*IRX3*	-
		14	9,816,993–10,025,165	*EBF2*	-
	CD	1	183,915,339–184,140,122	*SAMD4A*	Intramuscular fat content
		1	184,499,914–184,550,020	*DLGAP5*	Intramuscular fat content
		2	5,855,111–5,860,253	*CTSF*	-
		2	6,070,897–6,074,146	*CD248*	-
		7	97,614,707–97,624,273	*VRTN*	Intramuscular fat content et al.
		7	97,730,514–97,740,331	*NPC2*	Obesity Index
		7	98,065,323–98,074,822	*PROX2*	Obesity Index
		12	33,265,529–33,299,630	*SCPEP1*	-
10%	CD	3	8,863,695–8,871,564	*SERPINE1*	Abdominal fat weight Backfat weight et al.
		7	98,065,323–98,074,822	*PROX2*	Obesity Index
		12	54,680,057–54,738,103	*GLP2R*	Intramuscular fat content
		12	55,190,429–55,278,539	*MYH4*	Intramuscular fat content
		12	55,347,087–55,375,353	*MYH3*	-
		12	55,438,196–55,454,590	*TMEM220*	Intramuscular fat content

*Chr, chromosome; Pos (bp), gene position in Ensembl; QTL, quantitative trait loci*.

#### The 10% Level

For the AD line, 196 individuals were selected for the top 10% and bottom 10% groups, respectively, and were used to perform selection signature analysis. The results showed that there were differences from the 5% level. Several significantly differentiated areas at the 5% level disappeared at the 10% level, which means that with an increase in the number of selected individuals for the same trait, the selection intensity within the same Duroc line decreased. Five selected markers were identified in the AD line, and seven annotated genes were identified in the selected regions, but there were no genes related to BFT or lipid traits. For the CD line, 210 individuals in the top 10% and bottom 10% groups were selected for analysis, respectively, and a total of 38 selected markers were found, including 72 annotated genes, among which *SERPINE1, PROX2, GLP2R*, etc., were related to lipid metabolism (Figures 2E–H; Table 1). In addition, in the AD line, two overlapping selected markers and one gene *ATP8A1* were identified from both the 5 and 10% levels. *ATP8A1* participates in catalyzing the hydrolysis of ATP coupled to the transport of amino phospholipids from the outer to the inner leaflet of various membranes and ensures the maintenance of the asymmetric distribution of phospholipids (17). In the CD line, there were 18 overlapping selected markers and 19 genes from the two levels of which *PROX2* was related to the BFT trait and located in the obesity index QTL region (18) (Supplementary Table 5).

### Selection Signature of Loin Muscle Depth and Loin Muscle Area Within the American Duroc Line and the Canadian Duroc Line

#### The 5% Level

**Figure 3:**
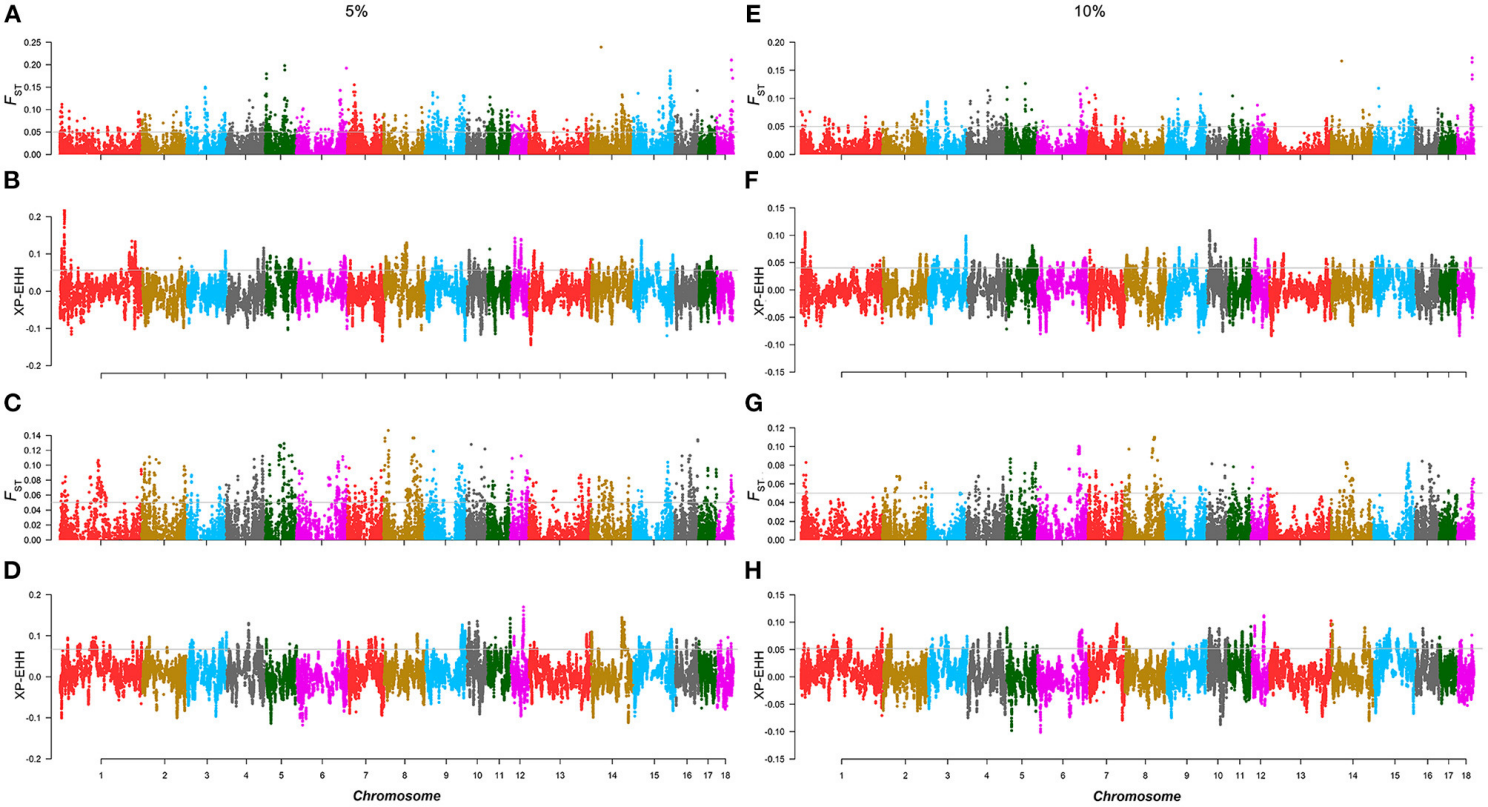
Selection signature detection for LMD and LMA in the 5 and 10% level. **(A,C,E,G)** are the Manhattans plots of *F*_ST_ of AD and CD lines in the 5 and 10% level. The gray lines denote threshold lines (*F*_ST_ = 0.05). **(B,D**,**F,H)** are the Manhattan plots of XP-EHH of AD and CD lines in the 5 and 10% level. The gray lines denote threshold lines (XP-EHH_5%AD_ = 0.057, XPEHH_5%CD_ = 0.067, XP-EHH_10%AD_ = 0.040, XP-EHH_10%CD_ = 0.052).

Similar to the above strategy, according to the rank of the TBV estimated from LMD and LMA, two 5% level groups were used to detect selection signatures in the two lines, respectively. For the AD line, we found 71 selected markers, and 130 genes were identified. GO and KEGG enrichment analyses were performed for these genes, among which *WNT10B, TLR2, PITX3*, and *SGCD* were involved in muscle tissue regulation and muscle development (Supplementary Figure 6). Similarly, the same method was performed in the CD line, and the results showed that 103 selected markers and 97 genes were identified. GO and KEGG enrichment analysis (Supplementary Figure 7) showed that *TMOD3, NEGR1*, and *PITX2* were associated with the trait (Figures 3A–D; Table 2).

**Table 2:** Candidate genes for loin muscle depth (LMD) and loin muscle area (LMA) in the 5% level analysis.

**Level**	**Line**	**Chr**	**Pos (bp)**	**Candidate gene**	**QTL**
5%	AD	5	14,997,381–15,013,919	*WNT10B*	Average backfat thickness et al.
		8	75,411,991–75,446,850	*TLR2*	-
		14	113,230,965–113,241,360	*PITX3*	-
		16	66,452,416–66,887,924	*SGCD*	-
	CD	1	119,862,244–119,948,883	*TMOD3*	-
		6	140,780,452–141,647,813	*NEGR1*	-
		8	111,698,664–111,723,298	*PITX2*	Drip loss et al.

*Chr, chromosome; Pos (bp), gene position in Ensembl; QTL, quantitative trait loci*.

#### The 10% level

Similar to the results of the 10% level analysis in BFT, the regions of significant differentiation in the 5% level analysis of LMD and LMA disappeared in that of the 10% level. For the AD line, a total of 22 selected markers were found, and 30 genes were identified, but none of these genes were related to muscle traits. As for the CD line, 58 selected markers and 23 annotated genes were identified, of which only *SLC44A5* was related to muscle development (Figures 3E–H). In addition, in the AD line, 6 overlapping selected markers and eight genes were identified from both the 5 and 10% levels, while a total of 39 overlapping selected markers and eight genes were identified in the CD line (Supplementary Table 6).

## Discussion

In this study, we conducted genomic analyses of 3,770 American Duroc pigs and 2,098 Canadian Duroc pigs to dissect the genetic differences and potential selection genes of growth traits in the two Duroc pig populations. Our results showed that the BFT in the CD line was higher than that of the AD line, while the LMD and LMA were lower than those of the AD line, which was consistent with the description of Wang et al. (1). Here, we think that the genetic differences between the two lines account for the differences in these traits. Therefore, we performed PCA (3), NJ-tree analysis, and LD decay analysis in the two lines, and the results showed that the AD and CD lines were clearly divided into two separate groups, with multiple branches within the AD line. The results of LD decay and *F*_*ROH*_ indicated that the CD line exhibited higher inbreeding. According to the differences in traits, we hypothesized that the genetic differences between the two lines may be caused by a combination of natural selection and artificial selection based on different breeding criteria, which resulted in phenotypic differentiation. Therefore, we performed selection signature analysis to detect the different selected genes in the two lines. The results showed that a series of genes selected in the AD line were enriched in the gland development pathway, while the genes were mainly involved in immune-related pathways in the CD line.

Many effects can affect quantitative traits, among which additive effect can be stably inherited by offspring. The EBV can be used for early selection, and even before the individual is born, the breeding value of the offspring can be predicted according to the performance of the two parents determined by the breeding plan. The AD and CD lines are commercial populations. In response to different breeding needs, selective breeding based on EBV ranking has been widely used in business (19). The individuals with better performance in traits in the same line can be retained; otherwise, they will be eliminated. Therefore, we adopted EBV and divided extreme individuals according to the ranking of EBV for analysis in this study, which is in line with actual production needs and patterns.

To reveal the genetic mechanism of growth traits in different lines, we divided different gradient levels to perform selection signature analysis. For the *BFT, IRX3*, and *EBF2* related to fat metabolism were identified in the AD line at the 5% level. *IRX3* is a functional long-range target of obesity-associated variants within *FTO, IRX3*-deficient mice reduces body weight by reducing fat mass and increasing basal metabolic rate and browning of white adipose tissue (20), and *EBF2* promotes the recruitment of beige adipocytes in white adipose tissue and protects animals against obesity (21). Seven genes including *SAMD4A, DLGAP5, VRTN, NPC2, PROX2, CD248*, and *SCPEP1* were identified in the CD line. Among these genes, *SAMD4A, DLGAP5*, and *VRTN* located in the IMF content QTL region (22–24), and *NPC2, PROX2* located in the obesity index QTL region (18). A missense mutation in the *CTSF* was significantly associated with average day gain, lean meat percentage, BFT, and feed conversion efficiency according to the study by Russo et al. (25). *CD248* is a sensitive marker of adipocyte function, increased expression of which leads to disturbances in glucose metabolism and ectopic deposition of lipids (26). *SCPEP1* regulates body fat content and is correlated with IMF deposition in pigs (27). However, at the 10% level, no trait-related genes were identified in the AD line. In contrast, *GLP2R, MYH4, TMEM220, SERPINE1*, and *MYH3* were identified in the CD line. *GLP2R, MYH4*, and *TMEM220* are located within the IMF content QTL region (23, 28), and *SERPINE1* is located in the QTL regions of abdominal fat weight, backfat weight, and subcutaneous fat, respectively (29). Besides, *MYH3* is a causal gene for the ratio of muscle fiber type, IMF content, and fat formation in pigs and mice (30).

We then performed selection signature analysis for LMD and LMA based on their TBV. At the 5% level, we identified *WNT10B, TLR2, PITX3*, and *SGCD* related to skeletal muscle development and repair in the AD line. *WNT10B* is involved in the *Wnt* signaling pathway and associated with skeletal muscle developmental regulation and regeneration (31). *TLR2* controls skeletal muscle repair mechanisms following different forms of injury (32). *PITX3* is widely expressed in skeletal muscles and promotes myogenic differentiation of muscle satellite cells (33). *SGCD* is a muscular dystrophy protein-related glycoprotein and abundantly expressed in skeletal and cardiac muscles (34). In the CD line, *TMOD3, NEGR1*, and *PITX2* were identified. *TMOD3* is involved in the regulation of actin and skeletal muscle contractions (35). *NEGR1* mediates neural cell communication and synapse formation, and deletion of this gene leads to increased adiposity and decreased muscle quality in mice (36). *PITX2* is involved in the regulation of skeletal muscle tissue development and animal organ morphogenesis (37). However, at the 10% level, we did not find trait-related genes in the AD line, but among the 30 genes obtained from the CD line, *SLC44A5* was found and fell within the QTL region of muscle fiber diameter (38).

In conclusion, population genetic analysis based on large samples showed that there was significant genetic differentiation between Duroc pigs with different genetic backgrounds in this study, which was also reflected in traits of different lines, such as the CD line with higher BFT, and the AD line with higher LMA and LMD. Selection signature detection between the AD and CD lines showed that there were different selective regions in the two lines. For the same line, we carried out selection signature detection at different levels based on EBV of BFT, LMD, and LMA phenotypes, and a series of genes associated with the three traits were identified, further illustrating the complexity of the genetic mechanism of quantitative traits. This study reveals the genetic differences between different lines of Duroc pigs after strong artificial selection and provides a reference for selecting different lines of Duroc pigs as sires for different needs.

## Data Availability Statement

Publicly available datasets were analyzed in this study. This data can be found here: https://doi.org/10.6084/m9.figshare.8019551.v1.

## Ethics Statement

The animal study was reviewed and approved by South China Agriculture University.

## Author Contributions

ZW and JY proposed the idea for the study. DL performed the bioinformatics analysis and wrote the paper. MH directed the analyses and revised the article. ZZ, RD, TG, LH, EZ, ZL, and GC collected the samples and recorded the phenotypes. ZW and GC contributed the materials. All authors reviewed and approved the manuscript.

## Funding

This study was supported by the National Natural Science Foundation of China (Grant No. 31972540), the Natural Science Foundation of Guangdong Province (Grant Nos. 2018B030313011 and 2020A1515111103), the Local Innovative and Research Teams Project of Guangdong Province (Grant No. 2019BT02N630), and the Guangdong Province Rural Revitalization Strategy Special Project (Grant No. 200-2018-XMZC-0001-107-0145).

## Conflict of Interest

The authors declare that the research was conducted in the absence of any commercial or financial relationships that could be construed as a potential conflict of interest.

## Publisher's Note

All claims expressed in this article are solely those of the authors and do not necessarily represent those of their affiliated organizations, or those of the publisher, the editors and the reviewers. Any product that may be evaluated in this article, or claim that may be made by its manufacturer, is not guaranteed or endorsed by the publisher.
