# Supplementary material for: Genomic Analyses Revealed the Genetic Difference and Potential Selection Genes of Growth Traits in Two Duroc Lines
Source: Front Vet Sci. 2021 Sep 7;8:725367. doi: 10.3389/fvets.2021.725367 (PMC8453014; doi:10.3389/fvets.2021.725367)
Supplement: Supplementary file 1 [file Data_Sheet_1.docx]

Supplementary Material

# Supplementary Figures and Tables

##
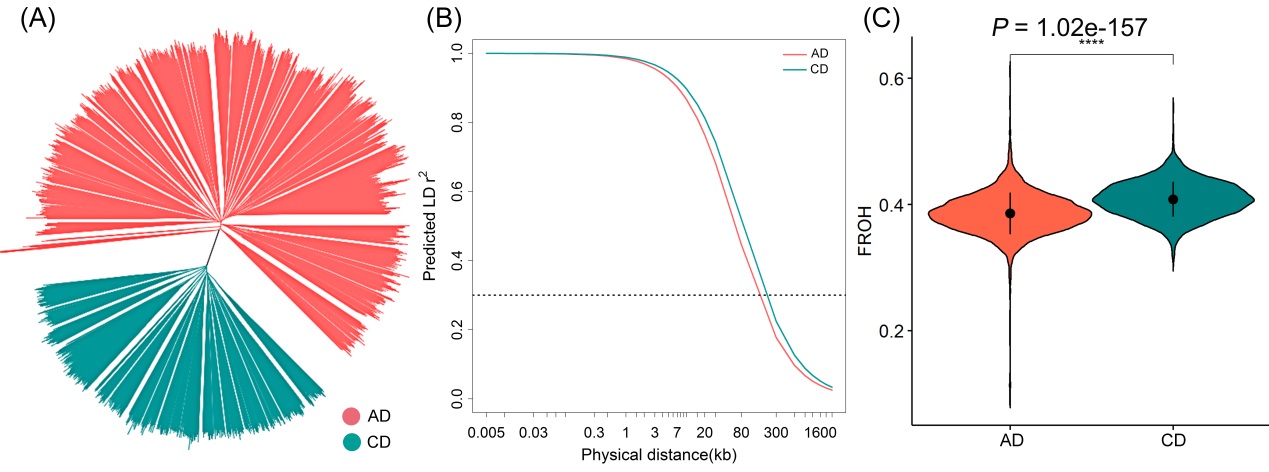
Supplementary Figures

**Supplementary Figure 1.** Population structure. (A) Neighbor-joining (NJ) phylogenetic tree (NJ-tree). (B) Linkage disequilibrium (LD) decay. The black dotted line represents the LD threshold (r^2^ = 0.3). When r^2^ =0.3, the physical distances of AD line is 150 kb and that of CD line is 202 kb. (C) Distribution of runs of homozygosity inbreeding coefficients within each line.

**Supplementary
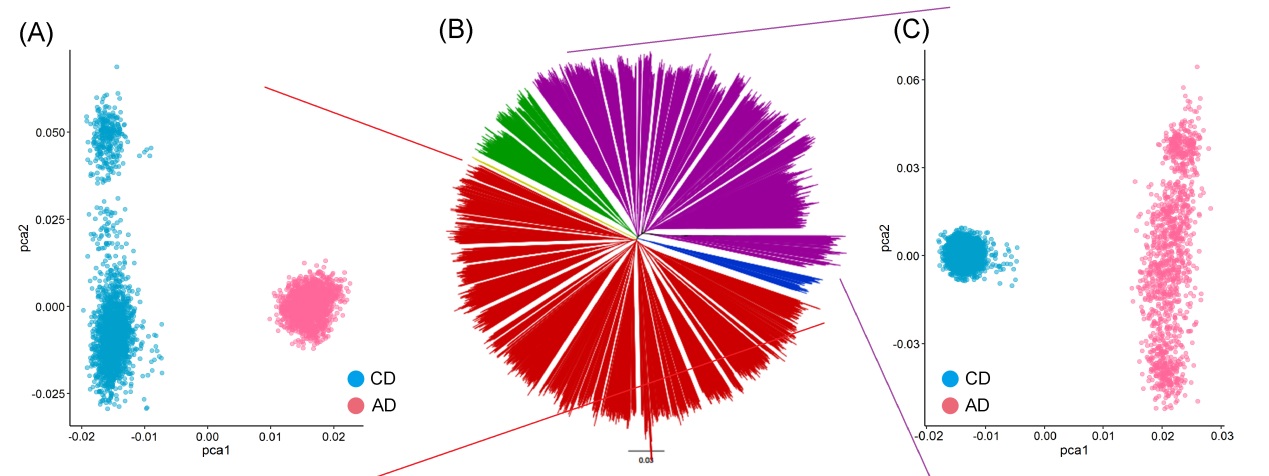
Figure 2.** Population structure of the AD. (A) Principal component analysis (PCA) between 1969 individuals in the red part of NJ-tree and the CD line. (B) Neighbor-joining (NJ) phylogenetic tree (NJ-tree) of the AD line. (C) PCA between 1314 individuals in the purple part of NJ-tree and the CD line. The 1969 individuals in the red part were selected for subsequent analysis.

**Supplementary
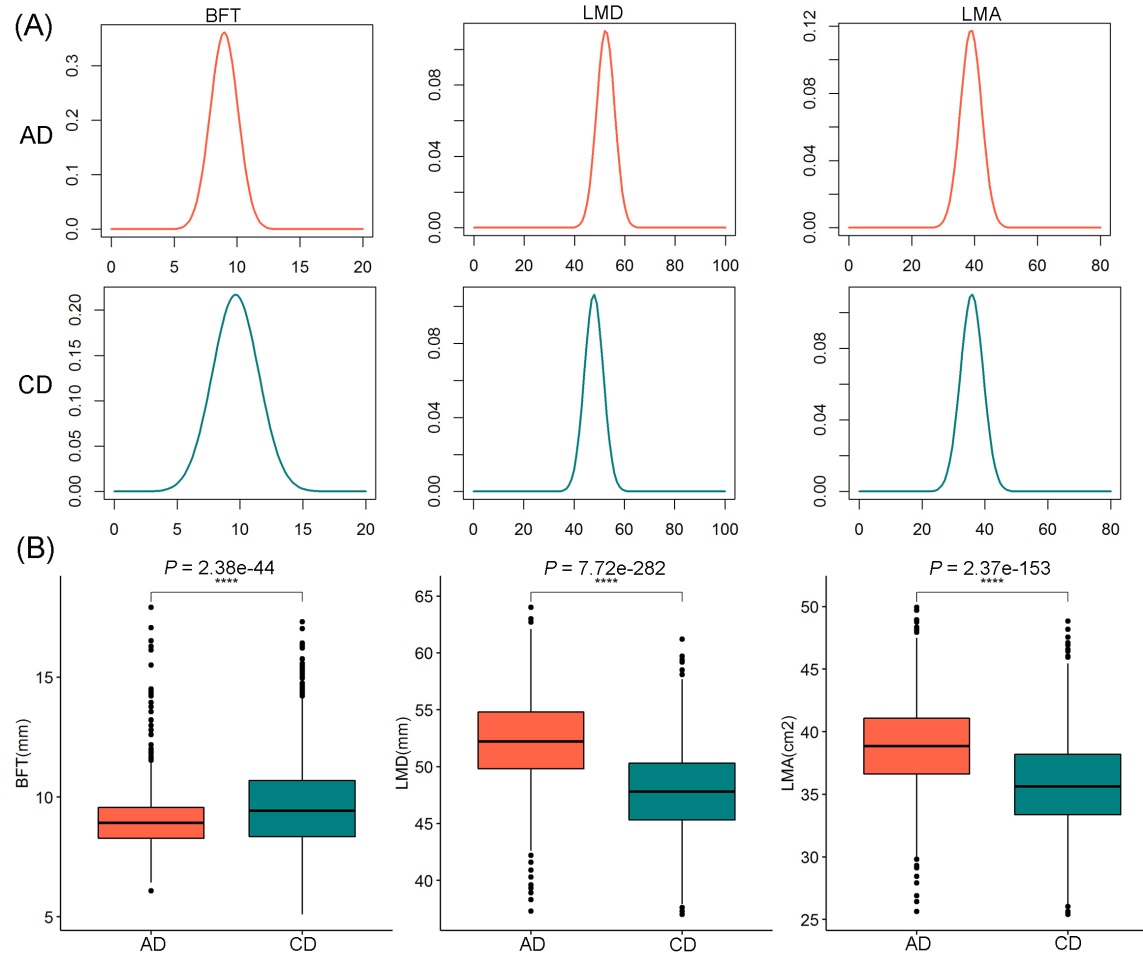
Figure 3.** Descriptive statistics of growth traits. (A) Normal distribution test for various growth traits. (B) Box plot of statistics of backfat thickness (BFT), loin muscle depth (LMD) and loin muscle area (LMA).

**Supplementary
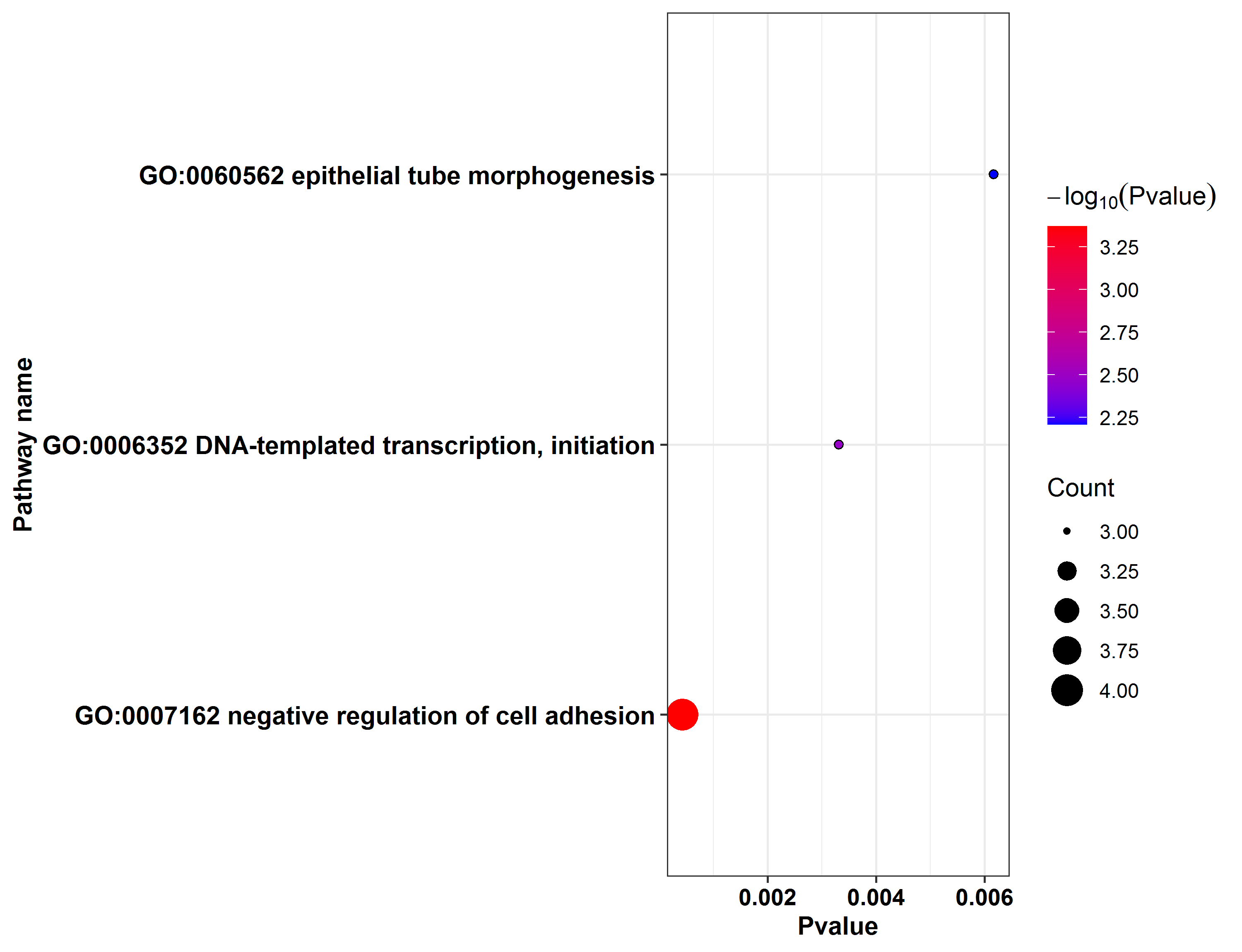
Figure 4.** The bubble chart of Gene Ontology (GO) terms and Kyoto Encyclopedia of Genes and Genomes (KEGG) pathways of the selected genes for BFT in the 5% level of AD line.

**Supplementary
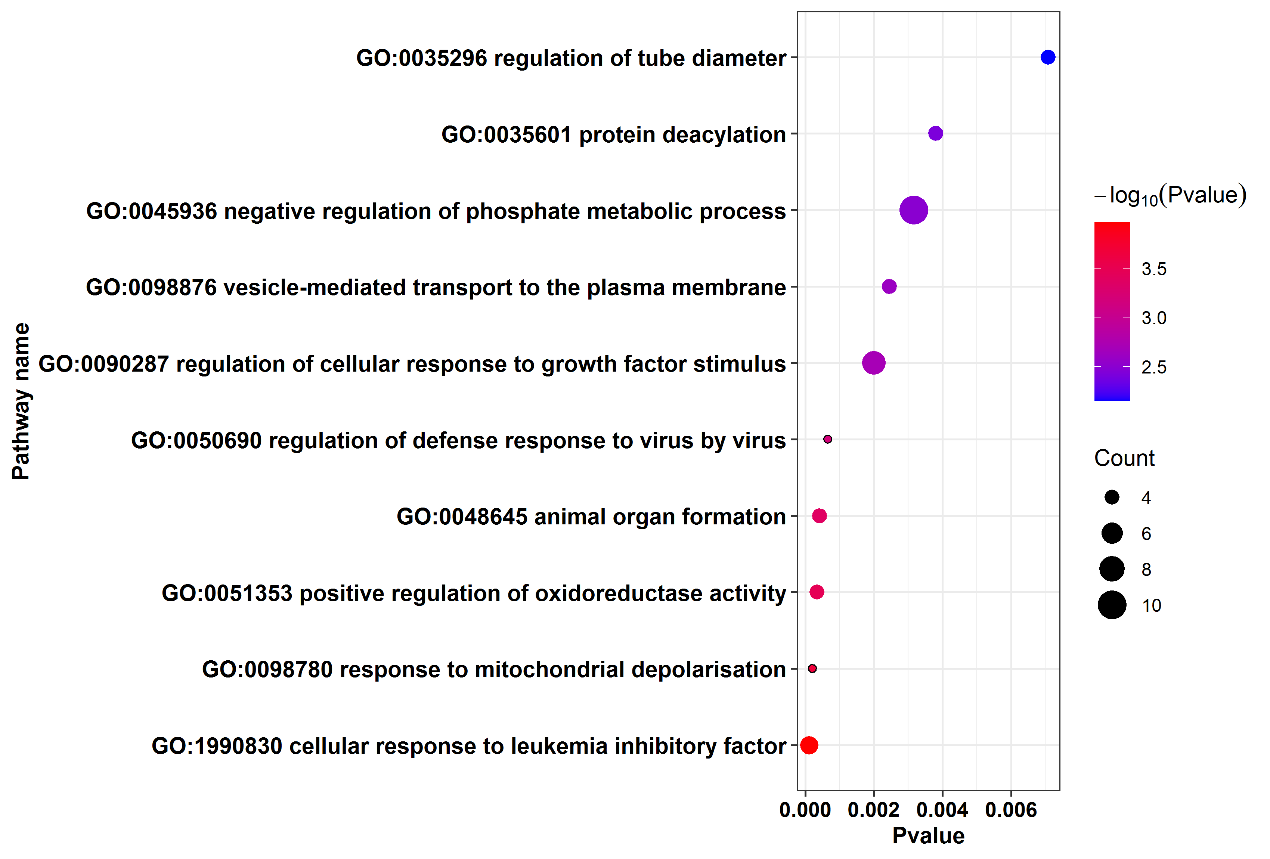
Figure 5.** The bubble chart of Gene Ontology (GO) terms and Kyoto Encyclopedia of Genes and Genomes (KEGG) pathways of the selected genes for BFT in the 5% level of CD line.

**Supplementary
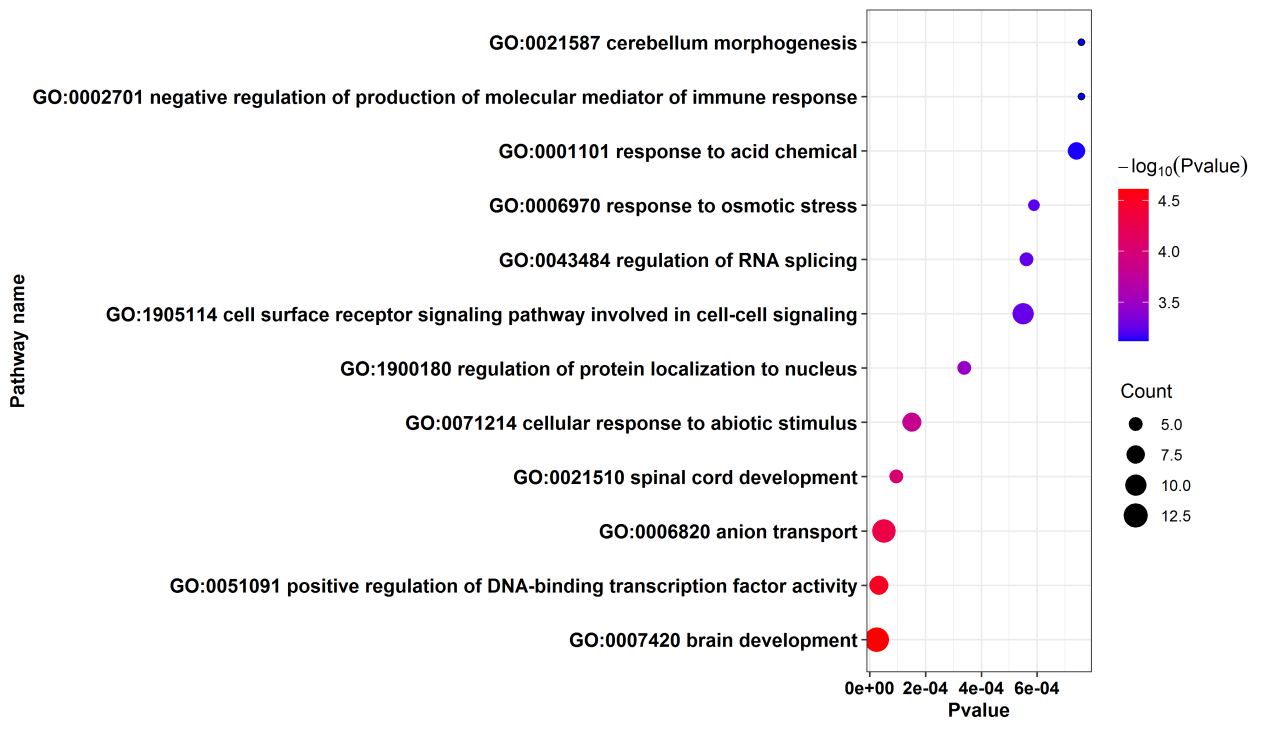
Figure 6.** The bubble chart of Gene Ontology (GO) terms and Kyoto Encyclopedia of Genes and Genomes (KEGG) pathways of the selected genes for LMD and LMA in the 5% level of AD line.

**Supplementary
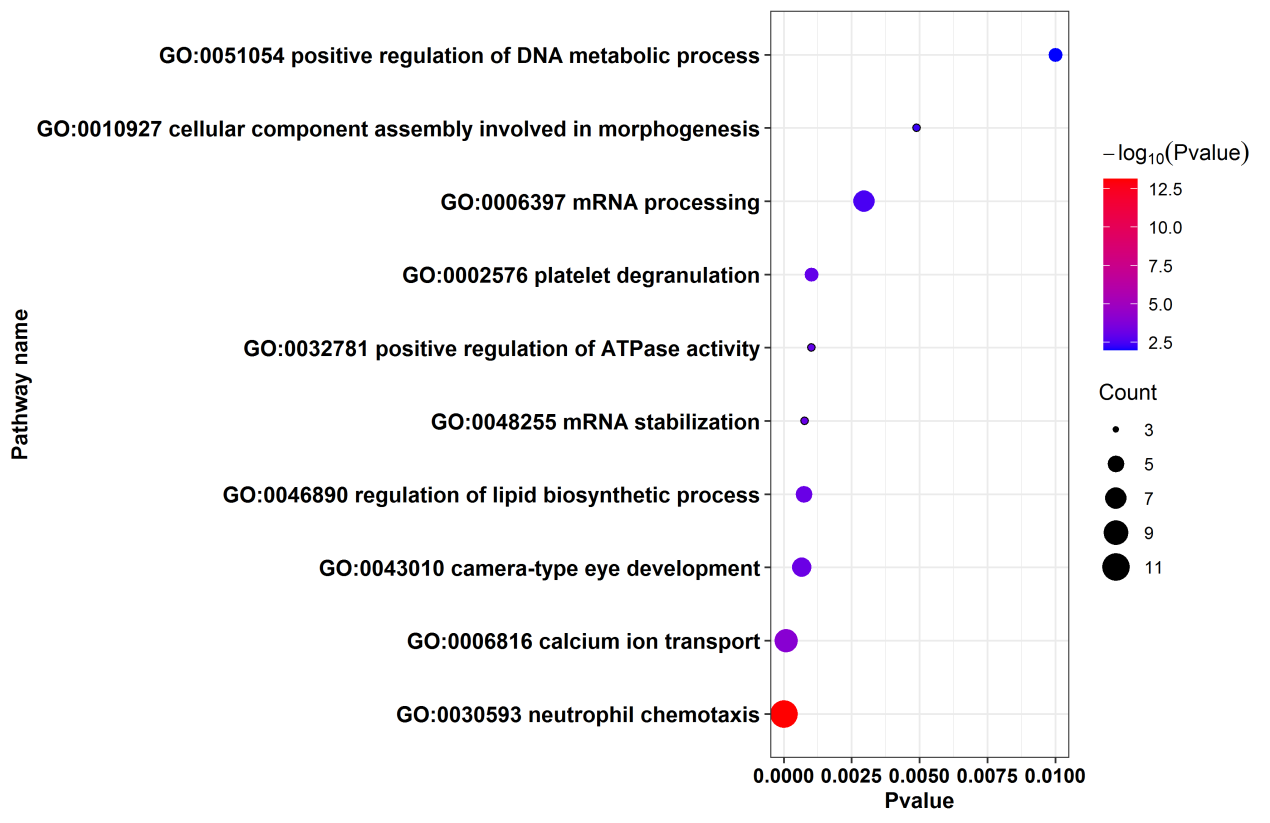
Figure 7.** The bubble chart of Gene Ontology (GO) terms and Kyoto Encyclopedia of Genes and Genomes (KEGG) pathways of the selected genes for LMD and LMA in the 5% level of CD line.

## Supplementary Tables

**Supplementary Table 1.** The division level of the fixed effects and random effects.

| **Line** | **Fixed effects** | | | | | **Random effects** |
| --- | --- | --- | --- | --- | --- | --- |
|  | **Sex** | **Farm** | **Year** | **Season** | **Parity** |  |
| AD | 2 | 10 | 5 | 4 | 10 | IID |
| CD | 2 | 1 | 3 | 4 | 8 |  |

Fixed effects division standards: Sex: divided into 2 levels (male and female). Farm: AD line divided into 10 levels, CD line was from the same farm and does not need to be divided. Year: AD line divided into 5 levels (born between 2013~2017), CD line divided into 3 levels (born between 2015~2017). Season: divided into 4 levels according to spring (March~May), summer (June~August), autumn (September~November), and winter (December~February). Parity: AD line divided into 10 levels, CD line divided into 8 levels.

**Supplementary Table 4.** The genes obtained from the selection signature between the AD and CD lines.

| **Line** | **Function** | **Gene** |
| --- | --- | --- |
| AD | Lipid metabolism | *FITM2, GPR55, CPT1A, HNF4A* |
|  | Reproduction | *CCNE2, CPEB2, ROBO1, CPT1A, HNF4A, LRP5, QDPR, SHH* |
|  | Growth | *R3HDML* |
| CD | Lipid metabolism | *SH2B2, ZNF385B* |
|  | Reproduction | *TCP11L1, WT1, CCDC73, VEGFA, CCL11, CCL2, SPRASA、SNORA70* |
|  | Growth | *DLK2, CSTB* |
|  | Immune system | *GTPBP2, ORAI2, ALKBH4, CRACR2A, CCL1, CD44, CSTB, MSH6, MSH2, PDXK, TMEM98, COMMD9, VEGFA, SPACA3* |

**Supplementary Table 5.** Overlapping SNPs and genes in the 5% and 10% level analysis of BFT

| **Line** | **Chr** | **SNP** | **Pos (bp)** | **Gene** | |
| --- | --- | --- | --- | --- | --- |
| AD | 8 | DRGA0008541 | 33580081 | *ATP8A1* | |
|  | 8 | ALGA0047524 | 33613427 | *ATP8A1* | |
| CD | 3 | H3GA0009277 | 35094559 | *-* | |
|  | 3 | H3GA0009279 | 35120951 | *-* |  |
|  | 5 | ASGA0027228 | 102107494 | *-* | |
|  | 7 | ASGA0035527 | 98089286 | *AREL1, FCF1, YLPM1, PROX2, DLST, RPS6KL1, EIF2B2, MLH3, ACYP1, ZC2HC1C, NEK9* | |
|  | 8 | ASGA0039617 | 109834853 | *-* | |
|  | 12 | ASGA0054167 | 33564945 | *AKAP1, MSI2* | |
|  | 12 | H3GA0034133 | 33577229 | *AKAP1, MSI2* | |
|  | 12 | MARC0072638 | 33662571 | *AKAP1, MSI2* | |
|  | 13 | ALGA0072897 | 174898503 | *-* | |
|  | 13 | WU_10.2_13_185433201 | 174939410 | *-* | |
|  | 13 | H3GA0037651 | 175387715 | *ROBO1* | |
|  | 13 | ASGA0059371 | 175453412 | *ROBO1* | |
|  | 13 | ALGA0072927 | 175488212 | *ROBO1* | |
|  | 13 | WU_10.2_13_186302899 | 175866327 | *ROBO1* | |
|  | 13 | WU_10.2_13_186977971 | 176572555 | *ROBO1* | |
|  | 14 | M1GA0019680 | 137239079 | *CLRN3, PTPRE* | |
|  | 15 | MARC0074677 | 18713489 | *-* | |
|  | 16 | ALGA0118313 | 20103323 | *ADAMTS12, SLC45A2, RAI14* | |

Chr: Chromosome. SNP: Single Nucleotide Polymorphism. Pos (bp): SNP position in Ensembl.

**Supplementary Table 6.** Overlapping SNPs and genes in the 5% and 10% level analysis of LMD and LMA.

| **Line** | **Chr** | **SNP** | **Pos (bp)** | **Gene** |
| --- | --- | --- | --- | --- |
| AD | 4 | H3GA0014519 | 121432780 | *-* |
|  | 4 | ALGA0028960 | 121496005 | *-* |
|  | 5 | WU_10.2_5_2006549 | 4651643 | *PRR5, RTL6, SHISAL1* |
|  | 9 | ASGA0042913 | 44054340 | *BUD13, ZPR1, APOA4* |
|  | 9 | MARC0030802 | 44393474 | *SIK3, PAFAH1B2* |
|  | 15 | WU_10.2_15_138765763 | 125112361 | *-* |
| CD | 6 | WU_10.2_6_128610471 | 138838325 | *FPGT, LRRIQ3* |
|  | 6 | ALGA0103459 | 139690809 | *-* |
|  | 6 | WU_10.2_6_129401618 | 139733199 | *-* |
|  | 6 | ALGA0036989 | 140086791 | *-* |
|  | 6 | WU_10.2_6_129862263 | 140250985 | *-* |
|  | 6 | ASGA0084764 | 140290906 | *-* |
|  | 6 | WU_10.2_6_129994069 | 140383033 | *-* |
|  | 6 | WU_10.2_6_130198007 | 140654006 | *NEGR1* |
|  | 6 | WU_10.2_6_130120168 | 140731062 | *NEGR1* |
|  | 6 | ALGA0037010 | 140767615 | *NEGR1* |
|  | 6 | ALGA0037015 | 140789137 | *NEGR1* |
|  | 6 | WU_10.2_6_130421506 | 140871782 | *NEGR1* |
|  | 6 | MARC0087895 | 140918839 | *NEGR1* |
|  | 6 | WU_10.2_6_130535063 | 140970557 | *NEGR1* |
|  | 6 | DRGA0006899 | 141005057 | *NEGR1, ZRANB2* |
|  | 6 | WU_10.2_6_130618252 | 141053645 | *NEGR1, ZRANB2* |
|  | 6 | WU_10.2_6_130699317 | 141134633 | *NEGR1, ZRANB2* |
|  | 6 | WU_10.2_6_130724101 | 141159400 | *NEGR1, ZRANB2* |
|  | 6 | WU_10.2_6_130768383 | 141203652 | *NEGR1, ZRANB2* |
|  | 6 | WU_10.2_6_130870598 | 141255839 | *NEGR1, ZRANB2* |
|  | 6 | ALGA0104761 | 141317111 | *NEGR1, ZRANB2* |
|  | 6 | WU_10.2_6_131071411 | 141416045 | *NEGR1, ZRANB2* |
|  | 6 | ALGA0117684 | 141548853 | *NEGR1, ZRANB2* |
|  | 6 | WU_10.2_6_131177159 | 141626290 | *NEGR1, ZRANB2* |
|  | 6 | WU_10.2_6_82457398 | 141634728 | *NEGR1, ZRANB2* |
|  | 6 | WU_10.2_6_131290611 | 141675802 | *NEGR1, ZRANB2* |
|  | 6 | ALGA0109995 | 141707663 | *NEGR1, ZRANB2* |
|  | 6 | ALGA0118586 | 141730151 | *NEGR1, ZRANB2* |
|  | 6 | WU_10.2_6_131388614 | 141773550 | *NEGR1, ZRANB2, PTGER3* |
|  | 6 | WU_10.2_6_131420561 | 141805479 | *NEGR1, ZRANB2, PTGER3* |
|  | 6 | MARC0036255 | 141914558 | *ZRANB2, PTGER3* |
|  | 6 | 6_131564469 | 141948827 | *ZRANB2, PTGER3* |
|  | 6 | ASGA0029626 | 142170580 | *PTGER3* |
|  | 6 | WU_10.2_6_131816487 | 142201251 | *PTGER3* |
|  | 6 | ASGA0098042 | 142358005 | *PTGER3, CTH* |
|  | 6 | WU_10.2_6_132010759 | 142396954 | *CTH, ANKRD13C* |
|  | 15 | ASGA0070932 | 121984795 | *-* |
|  | 15 | MARC0075123 | 122294134 | *-* |
|  | 15 | DRGA0015544 | 122364091 | *-* |

Chr: Chromosome. SNP: Single Nucleotide Polymorphism. Pos (bp): SNP position in Ensembl.
